# Accuracy of *Pf*HRP2 *versus Pf*-pLDH antigen detection by malaria rapid diagnostic tests in hospitalized children in a seasonal hyperendemic malaria transmission area in Burkina Faso

**DOI:** 10.1186/1475-2875-13-20

**Published:** 2014-01-13

**Authors:** Jessica Maltha, Issa Guiraud, Palpouguini Lompo, Bérenger Kaboré, Philippe Gillet, Chris Van Geet, Halidou Tinto, Jan Jacobs

**Affiliations:** 1 Department of Clinical Sciences, Institute of Tropical Medicine, Nationalestraat 155, B 2000, Antwerp, Belgium; 2 Centre for Molecular and Vascular Biology, University of Leuven, Leuven, Belgium; 3 IRSS/Clinical Research Unit of Nanoro (CRUN), Nanoro, Burkina Faso; 4 Paediatrics, University of Leuven, Leuven, Belgium

## Abstract

**Background:**

In most sub-Saharan African countries malaria rapid diagnostic tests (RDTs) are now used for the diagnosis of malaria. Most RDTs used detect *Plasmodium falciparum* histidine-rich protein-2 (*Pf*HRP2), though *P. falciparum*-specific parasite lactate dehydrogenase (*Pf*-pLDH)-detecting RDTs may have advantages over *Pf*HRP2-detecting RDTs. Only few data are available on the use of RDTs in severe illness and the present study compared *Pf*-pLDH to *Pf*HRP2-detection.

**Methods:**

Hospitalized children aged one month to 14 years presenting with fever or severe illness were included over one year. Venous blood samples were drawn for malaria diagnosis (microscopy and RDT), culture and complete blood count. Leftovers were stored at −80 °C and used for additional RDT analysis and PCR. An RDT targeting both *Pf*HRP2 and *Pf*-pLDH was performed on all samples for direct comparison of diagnostic accuracy with microscopy as reference method. PCR was performed to explore false-positive RDT results.

**Results:**

In 376 of 694 (54.2%) included children, malaria was microscopically confirmed. Sensitivity, specificity, positive predictive value (PPV) and negative predictive value were 100.0, 70.9, 69.4 and 100.0%, respectively for *Pf*HRP2-detection and 98.7, 94.0, 91.6 and 99.1%, respectively for *Pf*-pLDH-detection. Specificity and PPV were significantly lower for *Pf*HRP2-detection (*p* <0.001). For both detection antigens, specificity was lowest for children one to five years and in the rainy season. PPV for both antigens was highest in the rainy season, because of higher malaria prevalence. False positive *Pf*HRP2 results were associated with prior anti-malarial treatment and positive PCR results (98/114 (86.0%) samples tested).

**Conclusion:**

Among children presenting with severe febrile illness in a seasonal hyperendemic malaria transmission area, the present study observed similar sensitivity but lower specificity and PPV of *Pf*HRP2 compared to *Pf*-pLDH-detection. Further studies should assess the diagnostic accuracy and safety of an appropriate *Pf*-pLDH-detecting RDT in field settings and if satisfying, replacement of *Pf*HRP2 by *Pf*-pLDH-detecting RDTs should be considered.

## Background

Malaria rapid diagnostic tests (RDTs) are currently rolled out in sub-Saharan Africa to fulfill the need of parasite based diagnosis *e.g.* the parasitological confirmation of malaria before start of treatment [1]. The operational characteristics of RDTs have been extensively evaluated for uncomplicated malaria [2] and the parasite-based diagnosis strategy has proven to be safe in uncomplicated malaria [3]. In contrast, only a few studies addressed the use of RDTs in children presenting with severe illness. Those performed reported low specificity of *Plasmodium falciparum* histidine-rich protein-2 (*Pf*HRP2)-detecting RDTs [4,5], which is most probably due to *Pf*HRP2 persistence after clearance of infection [6]. An alternative would be an RDT detecting *P. falciparum*-specific parasite lactate dehydrogenase (*Pf*-pLDH), which is more rapidly cleared from the bloodstream, but lower sensitivities compared to *Pf*HRP2 have been reported [4]. However recent evaluations of other *Pf*-pLDH-detecting RDT products have shown better performance, also at low parasite densities [7-9]. The RDT used by the national malaria control programme of Burkina Faso detects *Pf*HRP2, which is recommended by the World Health Organization (WHO) [10] and used in most sub-Saharan African countries. The current diagnostic algorithm in Burkina Faso recommends treatment of malaria in case of a positive test and search for other causes of disease when negative [11], but does not differentiate between severe and non-severe disease. The aim of this study was to compare *Pf*-pLDH to *Pf*HRP2-detecting RDTs in children presenting with severe febrile illness in a seasonal, hyperendemic, malaria transmission area.

## Methods

### Study site and population

A one-year survey (July 2012–2013) to assess proportions and incidence rates of invasive bacterial infections and severe malaria was performed in a rural area in the centre-west region of Burkina Faso. In this region there is seasonal hyperendemic malaria transmission and the estimated under-five mortality in 2010 was 142/1,000 live births [12]. Details of the study have been published elsewhere [13]. In summary, children (<15 years) presenting with axillary temperature ≥38.0 °C and/or clinical signs of severe illness who were admitted to the hospital or health centre were enrolled. Signs of severe illness included convulsions, altered consciousness, prostration, respiratory distress, shock, hypothermia and severe malnutrition. For the present study, children < one month of age were not considered.

### Sample collection

In all children enrolled blood culture was performed and ethylene diamine tetra-acetic acid (EDTA)-anticoagulated venous blood samples were drawn for malaria diagnosis (both microscopy and RDT). Laboratory analysis was performed in the clinical research unit of Nanoro (CRUN), located on the compound of the district hospital. Leftovers of EDTA blood samples were stored at 80 °C within a maximum of two hours after sampling until further analysis. Medical history, including previous anti-malarial treatment, and clinical examination were registered on standardized forms by trained study staff.

### Laboratory procedures

Thick blood films (TBF) were stained with Giemsa and assessed for the presence of *Plasmodium* parasites according to standard procedures [14]. Parasite density was expressed as asexual parasites per μl using the patient’s white blood cell (WBC) count. TBF was considered negative if no parasites were seen on 100 fields. Every slide was read by two experienced microscopists blinded to each other’s results and in case of discrepant results (positive *vs* negative, different *Plasmodium* species, difference in parasite density > Log10 or ratio >2 in case of parasite density ≤400/μl and >400/μl, respectively) by a third experienced microscopist.

The RDT SD Bioline Malaria Antigen P.f (Standard Diagnostics, Hagal-Dong, Korea), further referred to as SD50 (LOT 082160), is the RDT recommended by the national malaria control programme of Burkina Faso and detects the protein *Pf*HRP2. SD50 was performed on EDTA blood samples by trained CRUN laboratory staff before slides were read and within a maximum of two hours after sampling. Blood culture work-up and cerebrospinal fluid analysis was performed according to standard microbiological procedures as described previously [13].

### Malaria rapid diagnostic test evaluated

The RDT SD Bioline Malaria Antigen P.f (HRP2/pLDH) (Standard Diagnostics, Hagal-Dong, Korea), further referred to as SD90 (LOT RDT12002), is a three-band test consisting of a control line and two test lines targeting *Pf*HRP2 and *Pf*-pLDH, respectively. Good performance was reported in previous evaluations [15,16]. SD90 was performed on EDTA blood samples according to the manufacturer’s instructions except for replacement of the transfer device by a micropipette. From February to July (n = 276), SD90 was performed on fresh samples side-to-side to SD50 by CRUN laboratory staff. For the remaining samples (n = 420) SD90 was performed on stored samples at the end of the study period by the investigator.

In case of absence of the control line the test was considered invalid and repeated. Test line intensities were scored as negative, faint, weak, medium, or strong compared to the control line by a single observer who was blinded to the result of microscopy. After reading, photographs were taken.

### Monitoring and quality control

A selection of slides (5%) was sent to the Institute of Tropical Medicine (ITM) and again read by an expert microscopist whose results were considered conclusive. SD50 and SD90 were ordered at ITM Belgium and shipped to Burkina Faso where they were stored in a temperature-controlled room. The actual kit in use was stored in the parasitology laboratory. Temperature and humidity during shipment and storage were monitored using loggers (Ebro Electronic GmBH, Ingolstadt, Germany).

For discordant results between either SD50 or SD90 and microscopy, samples were retrieved from −80 °C storage and both RDTs were repeated by the investigator blinded to microscopy results. The result of the repeat testing was considered for analysis in case the first result was performed by CRUN staff. If both the first and repeat testing was performed by the investigator, the first result was considered. Photographs taken were verified to exclude clerical errors.

In order to compare *Pf*HRP2 results of SD50 and SD90, the RDTs were performed side to side on 10% randomly selected stored samples. For SD50, these 10% of stored samples were also compared to results obtained when prospectively performed on fresh samples.

### Additional analysis: polymerase chain reaction (PCR)

Real-time polymerase chain reaction (PCR) was performed in case of discordant results between microscopy and RDT. DNA was extracted from 200 ml whole blood using QIAamp DNA blood Mini kit (QIAGEN, Venlo, The Netherlands) or from TBF if needed [17]. DNA was amplified by a species-specific 18S rRNA real-time PCR (*P. falciparum/Plasmodium vivax*[18]), the *Plasmodium ovale/Plasmodium malariae* duplex was run simultaneously to confirm microscopically identified non-falciparum species.

### Data management, definitions and analysis

Data were double-entered in Epi info software (version 3.5.3). Statistical analysis was done with Stata 11 (Stata Corp, College Station, TX, USA). For the purpose of this study, microscopy was considered as the gold standard. Samples with asexual *P. falciparum* parasites seen on TBF (irrespective of parasite density and either as mono-infection or as mixed infection with *P. ovale* or *P. malariae*) were categorized as *P. falciparum* positive. The remaining samples, including samples with pure *P. falciparum* gametocytaemia, *P. ovale* or *P. malariae* as well as those with no parasites seen, were categorized as *P. falciparum* negative. For SD90, sensitivity and specificity were calculated for both *Pf*HRP2 and *Pf*-pLDH test lines. A visible test line in case of *P. falciparum* positive samples was considered true positive, no visible test line false negative. For *P. falciparum* negative samples, the absence of a visible test line was labelled as true negative, a visible line was labelled as false positive. Sensitivity, specificity and predictive values were calculated by age group and season and expressed with 95% CI. Differences were assessed for statistical significance using the Chi-square test, or Fisher exact test when appropriate, in case of independent data (*e.g*., comparison between the seasons) and with the McNemar test or paired proportion test for dependent data (*Pf*HRP2 *vs Pf*-pLDH).

### Ethical issues

The study was approved by the ethical committee of Burkina Faso and the University Hospital of Antwerp and by the institutional review board of ITM. Written informed consent was obtained from the parent or guardian of each child included.

## Results

### Study population and malaria microscopy results

During the one-year study period, 696 children aged one month to 14 years were included [13]. For two children (both *P. falciparum* positive with parasite densities of 62/μl and 35,194/μl, respectively), there was evidence of a sample switch during storage, both samples were excluded from analysis. The final collection consisted of 694 samples.

Demographic data of participants during the different seasons are shown in Table 1. Seasons were divided into rainy season (July to October, monthly microscopy positivity rate 67.9-87.9%), post-rainy season (November to February, monthly microscopy positivity rate 31.4-40.0%) and hot dry season (March to June, monthly microscopy positivity rate 2.9-17.2%).

**Table 1:** Demographic profile and diagnosis of children included during the different seasons

	**All year**	**Rainy season**	**Post-rainy season**	**Dry season**
Number	694	398	151	145
Age, median months (IQR)	20 (11–37)	21 (12–37)	16 (9–35)	17 (9–39)
Female sex, n (%)	310 (44.7)	176 (44.2)	79 (52.3)	55 (37.9)
Prior antimalarial treatment, n (%)	302 (43.5)	156 (39.2)	87 (57.6)	59 (40.7)
Microscopy *Pf* positive	376 (54.2)	304 (76.4)	55 (36.4)	17 (11.7)
*Pf* parasite density/μl, median	42,331	49,962.5	18,256	7,549
*Pf* parasite density/μl, range	25 - 702,500	62 - 702,500	99 - 259,685	25 - 112,465
Blood culture positive, n (%)	60 (8.7%)	19 (4.8)	27 (17.9)	14 (9.7)
Confirmed meningitis, n (%)	6 (0.9)	1 (0.3)	2 (1.3)	3 (2.1)
Co-infections	7 (1.0)	4 (1.0)	3 (2.0)	0

IQR = interquartile range, n = number, *Pf* = *P. falciparum.*

In 376 (54.0%) children, samples were *P. falciparum* positive, five of them had a mixed infection with *P. malariae* (n = 3) and *P. ovale* (n = 2). Median *P. falciparum* parasite density was 43,231.5/μl (25–702,500). Among the *P. falciparum* negative samples (318/694, 45.8%), two had *P. ovale* infection and 13 had pure *P. falciparum* gametocytaemia.

### Diagnostic accuracy of PfHRP2 and Pf-pLDH

**Figure 1:**
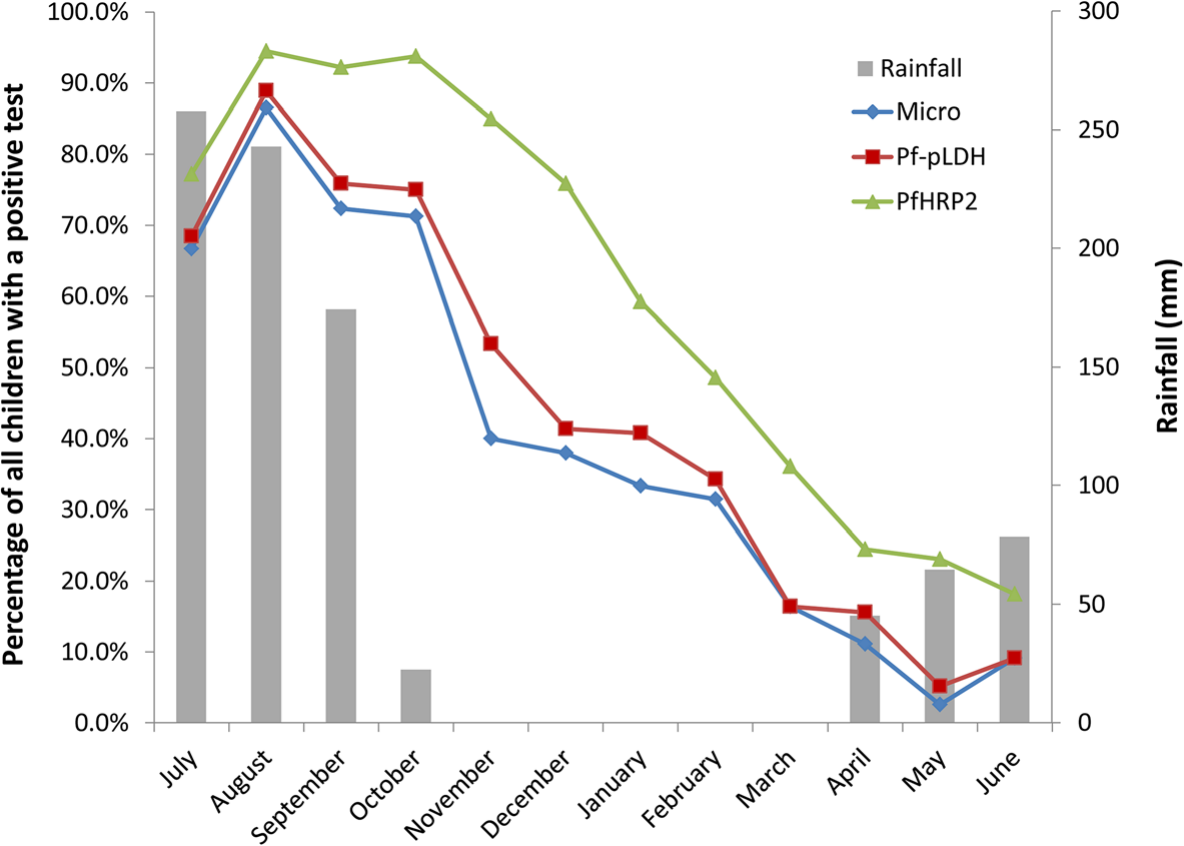
**Microscopy, *Pf*HRP2 and *Pf*-pLDH positivity rate by month.***Micro = microscopy positivity rate, Pf*HRP2 *= positivity rate of Pf*HRP2*, Pf-*pLDH *= positivity rate of Pf-*pLDH.

**Figure 2:**
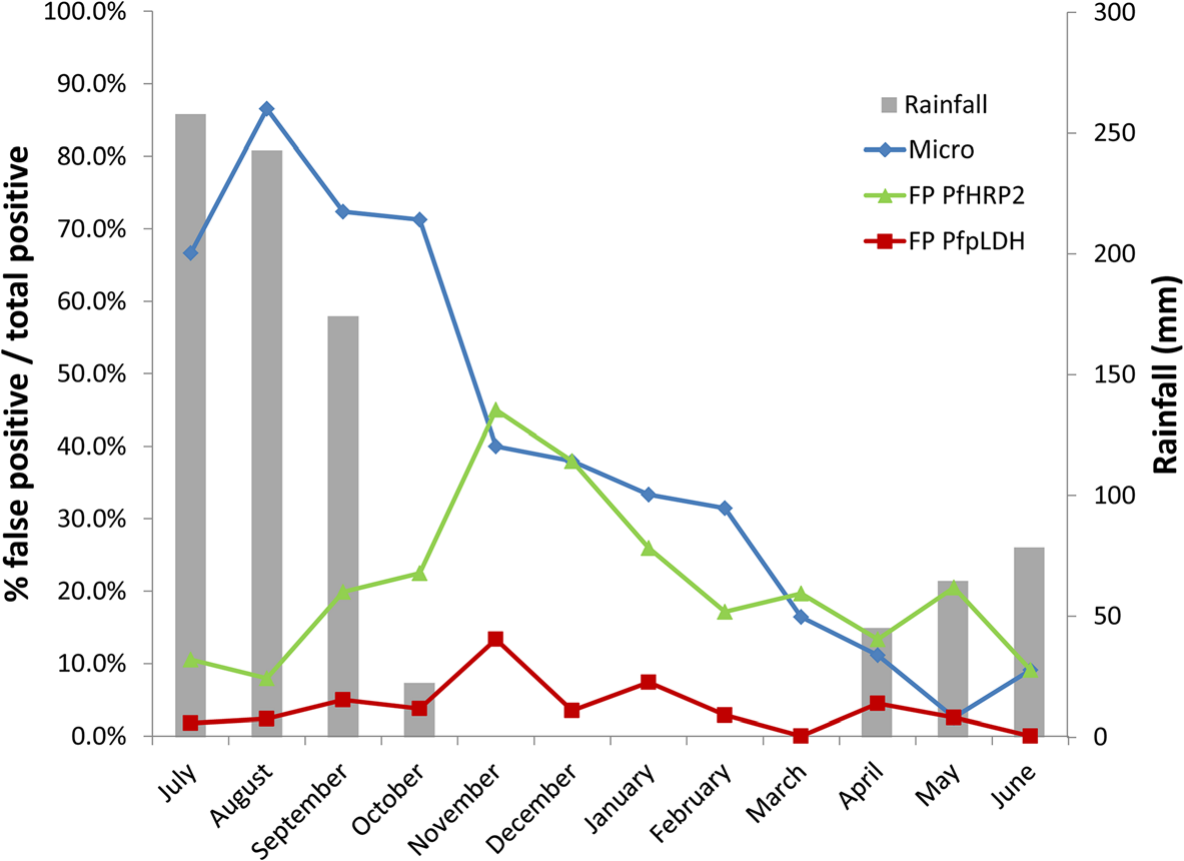
**False positive rapid diagnostic test results per month compared to microscopy positivity rate. ***Micro = microscopy positivity rate among all children included per month, FP PfHRP2 = % false positive PfHRP2 results among total positive PfHRP2 results, FP PfpLDH = % false positive Pf-pLDH results among total positive Pf-pLDH results.*

No invalid results were observed for SD90. *Pf*HRP2 and *Pf*-pLDH positivity rates are shown in Figure 1. Among the *P. falciparum*-positive samples, *Pf*HRP2 was positive in all samples while *Pf*-pLDH missed one (parasite density 62/μl, Table 2), resulting in an overall sensitivity of 100 and 99.5% for *Pf*HRP2 and *Pf*-pLDH, respectively (*p* = 1.0). Among the *P. falciparum*-negative samples, there were 139 and 29 false-positive *Pf*HRP2 and *Pf*-pLDH lines, respectively (Figure 2), corresponding to specificities of 56.3 and 90.9%, respectively (p <0.001). In 25 (7.9%) *P. falciparum*-negative samples both test lines were visible: they included eight samples with pure gametocytaemia. *Pf*HRP2 was positive in an additional 114 samples, including another four samples with pure gametocytaemia. False positive *Pf*-pLDH test lines in the absence of *Pf*HRP2 lines occurred in four samples of which two were *P. ovale* infection and one pure gametocytaemia.

**Table 2:** ***Pf***HRP2 and *Pf*-pLDH results according to parasite density

		***Pf*HRP2 pos**		***Pf*HRP2 neg**	
**Microscopy**	**Number**	***Pf*-pLDH pos**	***Pf*-pLDH neg**	***Pf*-pLDH pos**	***Pf*-pLDH neg**
1 - 100	3	2	1		
101 - 1,000	25	25			
1,001 - 10,000	66	66			
10,001 - 100,000	199	199			
> 100,000	83	83			
pure gametocytemia	13	8	4	1	
Microscopy negative	303	17	110	1	175
*P. ovale*	2			2	
Total	694	400	115	4	175

Neg = negative, pos = positive, *Pf*HRP2 = *P. falciparum* Histidine-rich protein-2, *Pf*-pLDH = *P. falciparum-*specific parasite lactate dehydrogenase.

Overall, positive predictive value (PPV) for *Pf*HRP2-detection (73.0%) was significantly lower compared to *Pf*-pLDH detection (92.8%, *p* <0.001) while negative predictive values (NPV) were similar (Table 3).

**Table 3:** **Diagnostic accuracy of *Pf*HRP2- compared to *Pf*-pLDH-detection**

	***Pf*HRP2**	***Pf*-pLDH**	***p*-value**
RDT pos, n (%)	515 (74.2)	404 (54.2)	
SE (95% CI)	100.0 (94.7 - 100.0)	98.7 (93.5-99.9)	1.0
Sp (95% CI)	70.9 (67.4 - 70.9)	94.0 (90.6 - 94.8)	< 0.001
PPV (95% CI)	69.4 (65.7 - 69.4)	91.6 (86.8 - 92.7)	< 0.001
NPV (95% CI)	100.0 (95.1 - 100.0)	99.1 (95.5 - 100.0)	1.0

N = number, NPV = negative predictive value, *Pf*HRP2 = *P. falciparum* Histidine-rich protein-2, *Pf*-pLDH = *P. falciparum-*specific parasite lactate dehydrogenase pos = positive, PPV = positive, predictive value SE = sensitivity, Sp = specificity.

### *Pf*HRP2 and *Pf-*pLDH performance by age group

*Pf*HRP2 specificity was significantly lower among children aged one to five years (39.6%) compared to children < one year (70.9%) or > five years (68.4%, *p* <0.001 for both), for *Pf*-pLDH differences among age groups were not significant (*p* = 0.053 and *p* = 0.134, respectively, Table 4). As the slide-positivity rate was highest among children one to five years of age, PPV for *Pf*HRP2 in this age group did not differ significantly from the others despite lower specificity (*p* = 0.428 and *p* = 0.255).

**Table 4:** **Diagnostic accuracy of *Pf*HRP2 *versus Pf*-pLDH by age group and by season**

	**Nr**	**Slide Pf pos (%)**	***Pf*HRP2**				***Pf*-pLDH**			
			**SE**	**Sp**	**PPV**	**NPV**	**SE**	**Sp**	**PPV**	**NPV**
			**(95**% **C.I.)**	**(95**% **C.I.)**	**(95**% **C.I.)**	**(95**% **C.I.)**	**(95**% **C.I.)**	**(95**% **C.I.)**	**(95**% **C.I.)**	**(95**% **C.I.)**
**Age (m)**										
1 – 11	194	39.7	100.0	70.9	69.4	100.0	98.7	94.0	91.6	99.1
			94.7-100.0	67.4-70.9	65.7-69.4	95.1-100.0	93.5-99.9	90.6-94.8	86.8-92.7	95.5-100.0
12 – 59	401	64.1	100.0	39.6	74.7	100.0	100.0	86.8	93.1	100.0
			98.3-100.0	36.6-39.6	73.5-74.7	92.5-100.0	98.4-100.0	84.0-86.8	91.6-93.1	96.7-100.0
≥ 60	99	42.4	100.0	68.4	70.0	100.0	100.0	94.7	93.3	100.0
			91.1-100.0	61.9-68.4	63.8-70.0	90.4-100.0	92.3-100.0	89.1-94.7	86.2-93.3	94.0-100.0
**Season**										
Rainy	398	76.4	100.0	35.1	83.3	100.0	99.7	85.1	95.6	98.8
			98.7-100.0	30.8-35.1	82.2-83.3	87.6-100.0	98.2-100.0	80.5-86.1	94.2-95.9	93.4-99.9
Post-rainy	151	36.4	100.0	46.9	51.9	100.0	100.0	87.5	82.1	100.0
			92.7-100.0	42.7-46.9	48.1-51.9	91.1-100.0	93.2-100.0	83.6-87.5	76.5-82.1	95.5-100.0
Hot dry	145	11.7	100.0	78.9	38.6	100.0	100.0	97.7	85.0	100.0
			78.7-100.0	76.1-78.9	30.4-38.6	96.4-100.0	81.7-100.0	95.2-97.7	69.5-85.0	97.5-100.0

Pf = *P. falciparum, Pf*HRP2 = *P. falciparum* Histidine-rich protein-2, *Pf*-pLDH = *P. falciparum-*specific parasite lactate dehydrogenase, pos = positive, SE = sensitivity, Sp = specificity, PPV = positive predictive value, NPV = negative predictive value.

### *Pf*HRP2 and *Pf-*pLDH seasonal performance

Sensitivity of *Pf*HRP2 and *Pf*-pLDH detection did not differ throughout the year (Table 4). *Pf*HRP2 specificity was significantly higher in the hot dry season (78.9%) compared to the rainy and post-rainy season (35.1 and 46.9%, respectively, both *p* <0.001). As slide positivity-rate was highest in the rainy season, PPV of *Pf*HRP2 was highest in the rainy season (83.3%), despite lowest specificity, and decreased to 51.9 and 38.6% in the other two seasons. Specificity of *Pf*-pLDH detection was in all seasons higher compared to *Pf*HRP2 (Table 4, *p* <0.001 for each season). Also for *Pf*-pLDH-detection PPV was highest in the rainy season, although differences were smaller: 95.6% in the rainy season compared to 82.1% (*p* <0.001) and 85.0% (*p* = 0.071) in the post-rainy and hot dry season, respectively.

### RDT positivity among children with invasive bacterial infections

Microscopy was positive in eight out of 64 (12.5%) children with invasive bacterial infections (IBI), *Pf*HRP2 and *Pf*-pLDH were positive in 33/64 (51.6%) and 12/64 (18.8%), respectively. As described previously [13] non-typhoid *Salmonella spp.* (NTS) were most frequently isolated from blood culture (21/60, 35.0%). *Pf*HRP2 was positive among 17/21 (81.0%) NTS, which was significantly more frequent compared to *Pf*-pLDH (6/21, 28.6%, *p* = 0.002) and microscopy (2/21, 9.5%, *p* < 0.001).

### RDT line intensity

For *Pf*-pLDH, 2.4% (nine/375) of true positive test lines was of faint intensity, for *Pf*HRP2 this was 0.5% (two/376). For the *P. falciparum* positive samples, the *Pf*HRP2 test line compared to the corresponding *Pf*-pLDH test line for the same sample was of stronger and weaker intensity in 179/376 (47.6%) and 31/376 (8.2%) samples, respectively. For seven samples with high parasite density (82,080-392,535/μl) *Pf*HRP2 was of weak intensity while *Pf*-pLDH was of strong intensity. Among the false positive test lines (excluding pure gametocytaemia), two/20 (10%) *Pf*-pLDH and 67/126 (53.2%) *Pf*HRP2 lines were of medium or strong intensity.

### RDT results of retesting and quality control

During the side-to-side comparison of *Pf*HRP2 of SD50 and SD90, no discordances between positive and negative results were observed and differences in line intensity were limited to one category. When comparing results of SD50 performed on fresh and stored samples, no differences in positive/negative results nor major differences in line intensities (more than one category) were observed, except for one originally strong result which was faint on repeat testing but for which clerical error could not be excluded.

### Further analysis of the false positive samples

**Figure 3:**
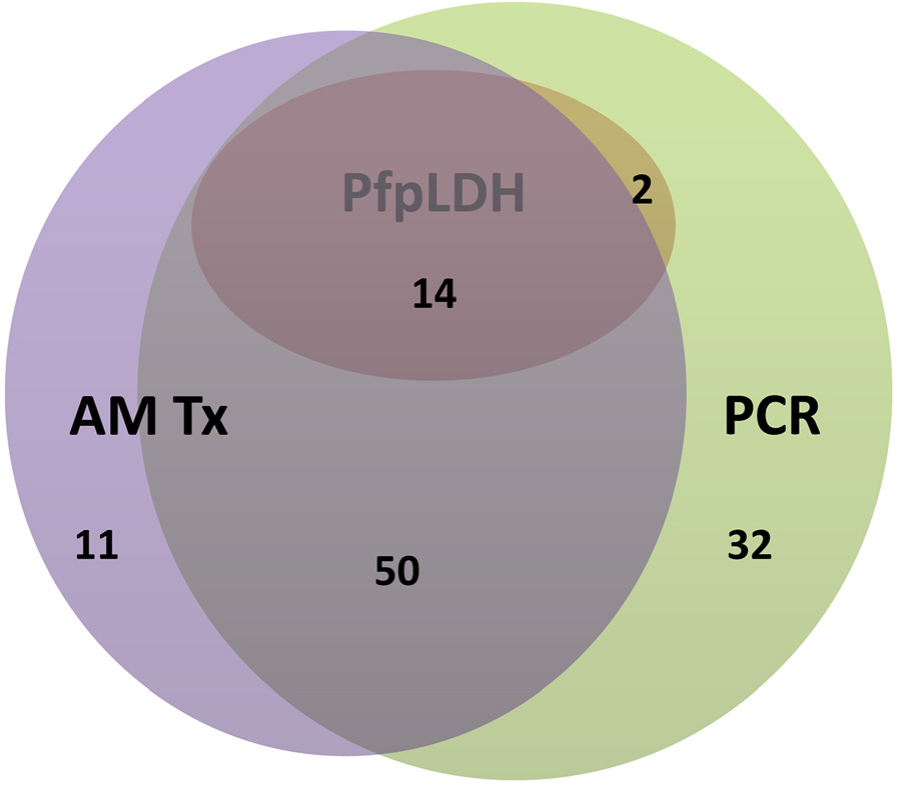
**Positive polymerase chain reaction and *Pf*-pLDH results and report of previous anti-malarial treatment for false-positive *Pf*** **HRP2 samples.***Only those samples with false positive PfHRP2 for which PCR was performed are displayed (n = 114). AM Tx = previous anti-malarial treatment, PfpLDH = positive Pf-pLDH test line, PCR = positive PCR result. For five children none of the aforementioned was positive.*

In 139/318 *P. falciparum*-negative samples the *Pf*HRP2 line was visible, including 12 samples with pure gametocytaemia. For the latter, ten/12 were obtained in children reporting previous anti-malarial treatment and eight/12 had a visible *Pf*-pLDH line. Figure 3 summarizes the results of PCR, *Pf*-pLDH and previous anti-malarial treatment for the false positive *Pf*HRP2 results (excluding pure gametocytaemia) for which PCR was performed (n = 114): *Pf*-pLDH was positive in 16 (14.0%) samples, PCR in 98 (86.0%) and previous anti-malarial treatment was reported in 75 (65.8%). For five samples, none of the aforementioned items was positive.

Among both *Pf*HRP2 and *Pf*-pLDH false positive results, report of previous anti-malarial treatment was significantly higher (66.2 and 86.2%, respectively) compared to microscopy positive samples (33.3%, *p* <0.001 for both).

## Discussion

The present study assessed the diagnostic accuracy of *Pf*HRP2 compared to *Pf*-pLDH antigen detection in children presenting with severe febrile illness in a rural area with seasonal malaria transmission. Both antigens had excellent sensitivity and similar negative predictive values, but *Pf*HRP2 had a lower specificity resulting in a significantly lower positive predictive value. Specificity was lowest in the rainy season, but due to the high malaria prevalence PPV was highest in the rainy season. The majority of false-positive *Pf*HRP2 lines were PCR positive and/or reported recent anti-malarial treatment, part of them were also *Pf*-pLDH positive or had pure gametocytaemia.

### Limitations

A number of limitations need to be considered. First, although the present study allowed reliable comparison between *Pf*-pLDH and *Pf*HRP2-detection, which was the study objective, the RDT evaluated is not a format that is likely to be used in field settings. However, the simultaneous side-to-side testing of SD90 and SD50 showed similar results, therefore data about *Pf*HRP2 performance can probably be extrapolated to the actual situation in Burkina Faso. Second, RDTs were performed and interpreted by an experienced investigator using a calibrated pipette, which may have generated higher sensitivities than would have been obtained under field conditions. Furthermore, 60% of SD90 was performed on stored samples, although samples had been stored for a maximum of one year and had not been thawed prior to testing; in addition, repeat testing on a subset of samples showed no difference in results when performed on fresh or stored samples.

### Sensitivity

So far, only two studies have evaluated the diagnostic accuracy of RDTs in children suspected of severe malaria, one in Mozambique and Tanzania [4] and another in Tanzania [5]. The former compared a *Pf*-pLDH with a *Pf*HRP2-detecting RDT: they observed a significant lower sensitivity for the *Pf*-pLDH-detecting RDT (88.0 *vs* 94.0%), especially at low (<1,000/μl) parasite densities. Of note however, the test used (Optimal-IT) is a multistep RDT with reported lower sensitivities compared to more recent one-step *Pf*-pLDH-detecting RDT products [8,19]. In the present study there was excellent sensitivity of *Pf*-pLDH detection, also at low parasite densities. Only one sample was missed by *Pf*-pLDH detection and this sample had a parasite density of 62/μl, which is below the detection threshold of routine microscopy [20].

Faint test lines, which are prone to be disregarded by health workers [21-23], only occurred in 0.5 and 2.4% of the true positive *Pf*HRP2 and *Pf*-pLDH lines, respectively. The co-presence of weak *Pf*HRP2 and strong *Pf*-pLDH lines at high parasite densities (n = 7), may be caused by the prozone effect (false negative/low test lines due to an antigen excess [24]), but was presently not further assessed. Only *Pf*HRP2-detecting RDTs are affected by the prozone effect, not *Pf*-pLDH [24,25]. The degree to which RDTs are affected by prozone is product dependent and in some products *Pf*HRP2 lines may be completely absent [24], leading to false negative results. The weak line intensities at high parasite densities are also of concern, as they may be considered as non-severe disease [23,26].

### Specificity

When interpreting specificity, several factors should be addressed: first, although in the present study slides were double-read by experienced microscopists, very low parasite densities may have been missed. Next, exclusive presence of gametocytes was considered as *P. falciparum* negative (as they do not cause clinical infection) but they produce *Pf*HRP2 and *Pf*-pLDH [27,28] explaining the apparent false positive results. For the purpose of this study PCR was not considered as reference method, because it may detect submicroscopic infections (reflecting asymptomatic carriage) which do not explain clinical symptoms [29]. The false positive *Pf*-pLDH lines observed in the present study can in part be explained by a (ongoing and partly) treated malaria infection from the previous days as *Pf*-pLDH becomes negative in a median of two to seven days after start of effective treatment [30,31]. This was presently supported by its association with a history of recent anti-malarial treatment. In addition, there were three false positive *Pf*-pLDH results for which both *Pf*HRP2 and PCR were negative. Possible explanations may be cross-reaction with pLDH produced by *P. ovale* (n = 2) or other interfering factors [32].

The interpretation of false positive *Pf*HRP2 lines is more complex. The most common cause of false positive *Pf*HRP2 results, especially in high-transmission areas, is *Pf*HRP2 persistence. Other possibilities, though rare, are non-specific bindings or interference with other immunological or infectious factors, such as the rheumatoid factor, hepatitis C, schistosomiasis, toxoplasmosis, dengue, leishmaniasis, Chagas disease and human African trypanosomiasis [19,33-36].

For the PCR negative samples, it can assumed that the false positive *Pf*HRP2 lines were due to past infection, approximately two to six weeks ago. For the PCR positive samples, the subsequent question arises whether the children were actually suffering from malaria at the time of sampling and had negative microscopy because of recently (<two days) started anti-malarial treatment (ongoing infection) [37] or whether it was a recently cleared infection with the child now suffering from another disease. Indeed, microscopy turns negative within one to two days after start of artemisinin-based combination therapy (ACT) [38], but the time of PCR to become negative after start of treatment has not yet been studied, although one study reported that upon completion of supervised ACT treatment a third of children were still positive by real time PCR [39]. As the proportion of positive PCR results among false positive *Pf*HRP2 samples was high in the present study, it can be assumed that at least part of the false-positive *Pf*HRP2 lines can be explained by ongoing and partly treated infection, especially in those samples that showed false positive *Pf*-pLDH results as well.

Prior use of anti-malarial treatment (either by self-medication or prescription) reflects real-life situation in malaria-endemic settings. To know if the child is actually suffering from malaria, an ideal RDT should be able to differentiate ongoing infection from a previously currently cured episode of infection, but *Pf*HRP2 is not capable of doing so. *Pf*-pLDH RDTs seem to be more promising in that respect as they turn negative in two to seven days, but future studies should assess their evolution over time after start of treatment.

### Influence of age and season on specificity

The low *Pf*HRP2 specificity in the rainy and post-rainy season compared to the dry season has been observed before [40] and may be explained by malaria infection in the weeks prior to enrolment, as malaria transmission is high in these months. In addition, children may have had an actual infection and been (partly) treated before enrolment, which also explains the decreased *Pf*-pLDH specificity in the rainy and post-rainy season. For children aged one to five years, specificity for *Pf*HRP2-detection was extremely low which may be ascribed to their high vulnerability to malaria, which was reflected by the high prevalence in this age group. The relationship has been observed before [5] while no such association was observed for *Pf*-pLDH detection, probably due to the more rapid clearance from the blood stream [41-43].

### What if treatment is based on RDT results?

In children with severe malaria it is crucial that the diagnosis is not missed: a negative RDT should safely exclude malaria. However, over diagnosis of malaria by diagnostic testing not only leads to a waste of anti-malarial drugs but also increases the risk of ignorance of other possible life-threatening diseases, such as invasive bacterial infections, which is especially true for *Pf*HRP2-detecting RDTs. Even though WHO mentions to look for other causes of severe illness (including IBI) in the case of a positive RDT, this strategy is not yet clearly implemented in the diagnostic algorithm of Burkina Faso [11] and may be overlooked in daily reality, especially since tools for diagnosis of other diseases are lacking.

### *Pf-*pLDH *versus Pf*HRP2-detecting RDTs

The present data adds to the debate about *Pf*-pLDH *vs Pf*HRP2-detecting RDTs. The previously reported lower sensitivity and lower heat stability of *Pf*-pLDH-detecting RDTs appeared to be product dependent [19]. Unlike *Pf*HRP2, *Pf*-pLDH-detection is not affected by the prozone effect [24], no gene deletions [15] or antigen polymorphisms [44-47] have been reported and it is rapidly cleared from the blood stream [41,42]. Especially because of its higher specificity, *Pf*-pLDH-detecting RDTs may be more useful in malaria-endemic areas. In addition it may be that *Pf*-pLDH-detecting RDTs are more cost-beneficial compared to presumptive diagnosis in high-transmission settings, which was not the case for *Pf*HRP2-detecting RDTs as concluded by Bisoffi *et al.*[48]. This needs however to be further evaluated.

### Future perspectives

To what extent can the present study findings be applied? First, an appropriate *Pf*-pLDH-detecting RDT should be selected and assessed for its diagnostic accuracy and robustness in field studies. Currently only few *Pf*-pLDH-detecting RDT products are available on the international market [32], and only one three-band RDT targeting *Pf*-pLDH and pan-pLDH and one two-band RDT targeting pan-pLDH fulfilled WHO criteria of good performance [49]. More emphasis should be on development and optimization of *Pf*-pLDH-detecting RDTs, and it should be evaluated how fast they become negative during anti-malarial treatment. If future field evaluations of *Pf*-pLDH-detecting RDTs are satisfying, the recommendation of WHO to use *Pf*HRP2-detecting RDTs in *P. falciparum-*endemic areas in sub-Saharan Africa [10] should be reconsidered. In the meantime, diagnostic algorithms should better highlight the possibility of invasive bacterial infections in spite of a positive RDT result.

## Conclusion

Among children presenting with severe febrile illness in a region with seasonal hyperendemic malaria transmission, similar sensitivity but lower specificity of *Pf*HRP2 compared to *Pf*-pLDH-detection was observed. Specificity of *Pf*HRP2 was lowest in the rainy season but PPV was highest in this season due to the high malaria prevalence. For each season and age group, the PPV of *Pf*HRP2 was lower compared to *Pf*-pLDH. Part of the apparent false-positive *Pf*HRP2 samples might however be due to parasite clearance after (incomplete or ongoing) treatment with anti-malarials at home or at the referring health centre. Further studies should assess the diagnostic accuracy and safety of an appropriate *Pf*-pLDH-detecting RDT in field settings and its capacity to distinguish ongoing malaria from recently cleared infections. If satisfying, replacement of *Pf*HRP2-detecting RDTs by *Pf*-pLDH-detecting RDTs should be considered.

## Abbreviations

ACT: Artemisinin-based combination therapy; CRUN: Clinical research unit of Nanoro; EDTA: Ethylene diamine tetra-acetic acid; IBI: Invasive bacterial infections; ITM: Institute of Tropical Medicine; NPV: Negative predictive value; NTS: Non-typhoid *Salmonella spp.*; Pan-pLDH: Pan *Plasmodium* lactate dehydrogenase; PCR: Polymerase chain reaction; PfHRP2: *P. falciparum* Histidine-rich protein 2; Pf-pLDH: *Plasmodium falciparum-*specific parasite lactate dehydrogenase; pLDH: Parasite lactate dehydrogenase; PPV: Positive predictive value; RDT(s): Rapid diagnostic test(s); TBF: Thick blood film; WHO: World Health Organization.

## Competing interests

The authors declare that they have no competing interests.

## Authors’ contributions

JM, CVG, TH, and JJ conceived and designed the study. JM, GI and BK supervised patient inclusion. JM, PL and PG performed the laboratory analyses. JM and JJ analysed the data and drafted the manuscript. All authors read and approved the final manuscript.
